# Did the early full genome sequencing of yeast boost gene function discovery?

**DOI:** 10.1186/s13062-023-00403-8

**Published:** 2023-08-14

**Authors:** Erwin Tantoso, Birgit Eisenhaber, Swati Sinha, Lars Juhl Jensen, Frank Eisenhaber

**Affiliations:** 1 Agency for Science, Technology and Research (A*STAR), Bioinformatics Institute (BII), 30 Biopolis Street #07-01, Matrix Building, Singapore, 138671 Republic of Singapore; 2 Agency for Science, Technology and Research (A*STAR), Genome Institute of Singapore (GIS), 60 Biopolis Street, Singapore, 138672 Republic of Singapore; 3 LASA – Lausitz Advanced Scientific Applications gGmbH, Straße Der Einheit 2-24, 02943 Weißwasser, Federal Republic of Germany; 4 European Bioinformatics Institute (EMBL-EBI), Wellcome Genome Campus, Hinxton, Cambridge, CB10 1SD UK; 5 Novo Nordisk Foundation Center for Protein Research, Faculty of Health and Medical Sciences, University of Copenhagen, Copenhagen, Denmark; 6 School of Biological Sciences, Nanyang Technological University, 60 Nanyang Drive, Singapore, 637551 Republic of Singapore

**Keywords:** *Saccharomyces cerevisiae*, Yeast, Gene function space, Uncharacterized genes, Gene function discovery rate, Protein function

## Abstract

**Background:**

Although the genome of *Saccharomyces cerevisiae* (*S. cerevisiae*) was the first one of a eukaryote organism that was fully sequenced (in 1996), a complete understanding of the potential of encoded biomolecular mechanisms has not yet been achieved. Here, we wish to quantify how far the goal of a full list of *S. cerevisiae* gene functions still is.

**Results:**

The scientific literature about *S. cerevisiae* protein-coding genes has been mapped onto the yeast genome via the mentioning of names for genomic regions in scientific publications. The match was quantified with the ratio of a given gene name’s occurrences to those of any gene names in the article. We find that ~ 230 elite genes with ≥ 75 full publication equivalents (FPEs, FPE = 1 is an idealized publication referring to just a single gene) command ~ 45% of all literature. At the same time, about two thirds of the genes (each with less than 10 FPEs) are described in just 12% of the literature (in average each such gene has just ~ 1.5% of the literature of an elite gene). About 600 genes have not been mentioned in any dedicated article. Compared with other groups of genes, the literature growth rates were highest for uncharacterized or understudied genes until late nineties of the twentieth century. Yet, these growth rates deteriorated and became negative thereafter. Thus, yeast function discovery for previously uncharacterized genes has returned to the level of ~ 1980. At the same time, literature for anyhow well-studied genes (with a threshold T10 (≥ 10 FPEs) and higher) remains steadily growing.

**Conclusions:**

Did the early full genome sequencing of yeast boost gene function discovery? The data proves that the moment of publishing the full genome in reality coincides with the onset of decline of gene function discovery for previously uncharacterized genes. If the current status of literature about yeast molecular mechanisms can be extrapolated into the future, it will take about another ~ 50 years to complete the yeast gene function list. We found that a small group of scientific journals contributed extraordinarily to publishing early reports relevant to yeast gene function discoveries.

**Supplementary Information:**

The online version contains supplementary material available at 10.1186/s13062-023-00403-8.

## Introduction

The choice of *S. cerevisiae* as the first fully genome-sequenced eukaryote in 1996 [1] was not by accident. Yeast is an extensively studied, excellent model organism for human biology since about a third of its genes has obvious orthologues in human [2] and it shares a very similar internal cell structure. Yeast is a beloved model for human cellular aging [3]. There are also many technical advantages such as (1) easy, cheap culturing and fast growth in the lab with doubling in < 2 h, (2) established techniques for genetic manipulation with simple gene knockouts in the haploid phase, (3) usage as model for meiotic cell division, and (4) the small fraction of non-protein-coding DNA in the genome.

In 1996, the bright future of yeast systems biology, “a new microbiology which … will enable the effective study of global physiological and metabolic problems involving a whole series of gene products” [4] was seen in near reach. Yet about a decade later, Pena-Castillo and Hughes [5] struggled with the disappointment that far more than 1000 protein-coding genes in yeast remain functionally uncharacterized. This implies that whole pathways and gene subnetworks are still in the dark and any systemic view must be limited if it is at all possible. Pena-Castillo and Hughes [5] tried to find an explanation why progress is so slow without finding an answer that satisfied themselves. Nevertheless, they extrapolated from their data that an almost complete gene function list for yeast should be achieved in ~ 2020.

At the time of writing this work (June 2023), the publication of the first yeast genome is more than a quarter of a century old. Yet, the Saccharomyces Genome Database [6–8] still lists 932 open reading frames (ORFs) coding for a “protein with unknown function. Clearly, the prediction of Pena-Castillo and Hughes [5] did not materialize.

In this work, we quantify the progress of gene function discovery over historical periods using a methodology applied previously to the human genome [9] as well as to the *Escherichia coli* (*E. coli*) pangenome [10]. We mapped the available scientific literature onto the yeast genome by using gene/protein/RNA names mentioned in the articles’ titles, abstracts, and full texts (if available). Since rarely an article talks only about one gene, we score each article for a given genomic entity with a fractional count as the ratio of references to a given gene in the text and the number of mentioning any gene. The sum of these scores for a given gene measures the available scientific literature in full publication equivalents (FPE), in terms of idealized articles reporting only about this one gene.

Our results not only show that there is a group of a few hundred “elite” yeast genes that command a disproportionate share of the total literature when about two thirds of the yeast genes appear seriously understudied. Hundreds of yeast gene names are not mentioned in any article. More importantly, we see that the rate of appearance of previously not mentioned yeast gene names in the literature dropped after the late 90-ies of the twentieth century and especially dramatically and permanently after ~ 2010, a development that Pena-Castillo and Hughes [5] could not foresee in 2007. The current rate of function discovery reports for previously not mentioned yeast genes is at the level of ~ 1980.

## Results

### Coverage of the *S. cerevisiae* gene function space by the available scientific literature

About 600,000 fractional counts for yeast genes (Additional file 3: Files 1 and 2) have been extracted from about 100,000 scientific texts (up to the qualifying date 19th of June 2023, for the collection of the text corpus). We reused the named entity recognition engine, the *S. cerevisiae* gene list (with a list of 6691 protein-coding genes), the keyword and synonym dictionary and the deduction rule system from the STRING database version 11.5 [11–13] for the automated mapping. Our methodology is described in more detail in the “Methods” section.

Table 1 presents the results of mapping the available literature on the genome of baker’s yeast. We find 6051 out of 6691 protein-coding genes mentioned at least once in a dedicated scientific article. The genome is extremely unevenly reflected in the literature. Just 235 “elite” genes, each with at least 75 FPEs (less than 4% of all protein-coding genes), the group of most intensively studied yeast genomic entities, are covered by ~ 45% of all relevant articles. Thus, every elite gene has ~ 0.2% of the relevant literature (~ 200 FPEs) on average.

**Table 1:** The number of *S. cerevisiae* genes as well as sums of literature scores in various FPE ranges

FPE score range	#Genes	Percentage of the total 6691 genes (%)	Literature score	Percentage of total score (%)	ƩGenes	Category
0	640	9.57	0.00	0.00	640	Not studied
0 < x < 1	1447	21.63	395.02	0.39	4120	Very understudied
1 ≤ x < 5	1714	25.62	4622.60	4.62		
5 ≤ x < 10	959	14.33	6886.99	6.88		
10 ≤ x < 15	530	7.92	6542.36	6.54	1062	Understudied
15 ≤ x < 20	307	4.59	5297.69	5.30		
20 ≤ x < 25	225	3.36	5014.21	5.01		
25 ≤ x < 30	152	2.27	4152.95	4.15	344	Moderately studied
30 ≤ x < 35	103	1.54	3355.06	3.35		
35 ≤ x < 40	89	1.33	3318.26	3.32		
40 ≤ x < 45	71	1.06	2999.29	3.00	290	Intensively studied
45 ≤ x < 50	61	0.91	2908.25	2.91		
50 ≤ x < 75	158	2.36	9670.73	9.67		
75 ≤ x < 100	66	0.99	5688.36	5.69	235	Very intensively studied
100 ≤ x < 500	155	2.32	26,730.43	26.72		
x ≥ 500	14	0.21	12,465.79	12.46		
Total	6691		100,048			–

This table lists the results of the automated mapping of publications onto the genome of baker’s yeast. We present the total number of genes in the respective FPE range at the time of this study (“#Genes”). We added a row for the 640 genes not specifically mentioned in any article about *S. cerevisiae* published until our cut-off date*.* Also, we computed the sum of the literature score for all genes in the respective FPE range (“Literature Score”). The total literature score 100,048 is equal to the total number of articles found with referencing a yeast gene in the main text, abstract or title. The FPE score range is further classified into six categories and the total number of genes in that category is provided (“ΣGenes”)

At the other end of the spectrum, our automated procedure did not find any article for 640 genes (~ 10% of the genome). Given the experience with the *E. coli* project [10], we think that a manual search might locate an article or two for some of them, which our conservative rule dictionary that is aimed at suppressing false-positive assignments due to ambiguous name usage might have overseen. So, the true number of genes without any article might be closer to 600. Yet, this is smaller than (though of a similar order of magnitude as) the number or 932 ORFs listed as coding for a “protein of unknown function” in the SGD database [6, 7].

For a further 4120 genes (~ 62% of the genome), the share of the total FPEs is just ~ 12%. The average literature share per gene is 0.003%. Thus, a gene in this category has just about 1.5% of the literature in average that an “elite” gene has.

### Changes of literature coverage of yeast genes in various historical periods

**Fig. 1:**
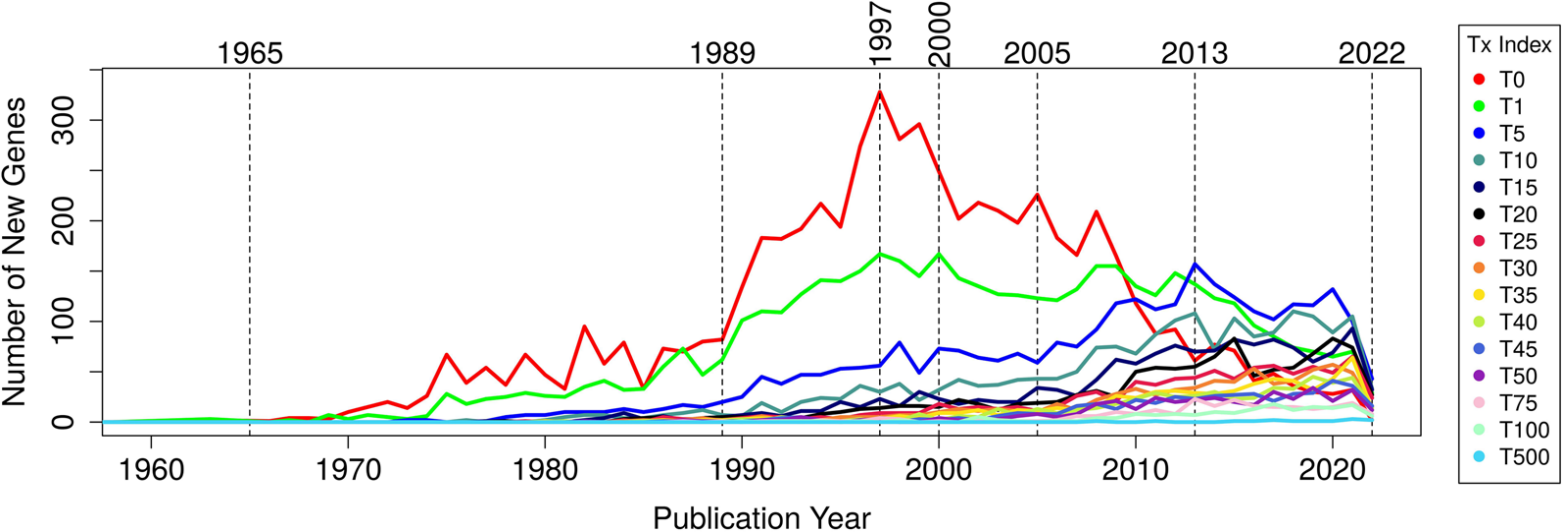
Gene function discovery rate from 1960 to 2022 for *S. cerevisiae*. The gene function discovery rate measured as the number of new genes first mentioned (T0) or crossing a specific threshold of aggregated FPEs (T1, T5, T10, …, T50, T75, T100 and T500) from year 1960 until year 2022

Figure 1 illustrates how many genes crossed certain FPE thresholds each year (see Additional file 3: File 3 for the respective data). For example, number T0 implies that how many genes have been mentioned in the literature for the first time in those years. The thresholds T1, T5, T10, …, T75, T100, and T500 mean that the respective genes each individually accumulated more than 1, 5, 10, …, 75, 100, or 500 FPEs in that year. Three historical periods regarding the gene function discovery dynamics can easily distinguished visually: (phase 1) a period of moderate growth until ~ 1990, (phase 2) a period of dramatic expansion until around 2000 and (phase 3) a drastic decline of new gene function discovery and refocus of research onto rather well-studied genes in the twenty-first century.

The dynamics is most expressed for T0. A steep increase up to the late 90-ties is followed by an abrupt drop 1997–2000 and another catastrophic one ~ 2010 with further, more moderate decrease thereafter. Whereas ~ 300 new yeast genes appeared in the literature every year in the late 90-ies, this number changed to dismal ~ 30 a quarter of a century later. The number of first reports on previously not mentioned genes in the past few years resembles the time ~ 1980 (Additional file 3: File 3).

Similarly, the curve for T1 (for T5) exhibits moderate growth until ~ 1990 (~ 2005), expedited increase until ~ 2000 (~ 2013) and dramatic decline thereafter. In the past few years (2020–2023), the number of new genes that reached T1 level is more like the status from the late 80-ies of last century. The T5 curve in 2020–2022 is more similar to the level from ~ 2008 (Additional file 3: File 3).

In contrast, the curves for T10–T35 show moderate growth until ~ 2007 and accelerated accumulation of additional new genes anytime thereafter. The respective annual numbers of genes climbing into categories T40–T500 grow steadily throughout the whole period of study.

This visual impression is supported by quantitative analyses with a linear regression model (Table 2). Whereas the slopes of the regression lines are by far the highest for T0, T1 and T5 (the literature coverage categories of uncharacterized or understudied genes) in phase 1, they get further boosted in phase 2 but decline in phase 3. For T5, T1 and, most dramatically, for T0, the slopes turn even negative indicating that the scale of new yeast gene function discovery has largely been reduced, even collapsed in the twenty-first century. For all other FPE threshold categories, there is a vigorous, increasing supply of new genes every year. Thus, we must conclude that research leading to more incremental improvement of functional characterization of generally well-studied genes is ongoing and rather expanding.

**Table 2:** The trend of literature coverage for *S. cerevisiae* genes in various FPE score thresholds

FPE score threshold	Phase 1—moderate growth				Phase 2—accelerated growth				Phase 3—decline			
	Slope	R^2^	*P*-value	Years	Slope	R^2^	*P*-value	Years	Slope	R^2^	*P*-value	Years
T0	3.58	0.73	1.03E−07	1965–1989 ↑	24.35	0.87	2.77E−04	1989–1997 ↑↑	−12.31	0.94	1.96E−15	1997–2021 ↓
T1	2.69	0.85	1.12E−10	1965–1989 ↑	7.90	0.83	3.95E−05	1989–2000 ↑↑	−3.65	0.62	1.30E−05	2000–2021 ↓
T5	2.69	0.89	1.02E−15	1975–2005 ↑	10.17	0.86	3.01E−04	2005–2013 ↑↑	−4.20	0.40	6.73E−02	2013–2021 ↓
T10	1.78	0.90	2.55E−14	1980–2007 ↑	2.72	0.48	4.27E−03	2007–2021 ↑↑	**–**	**–**	**–**	**–**
T15	1.13	0.83	1.32E−11	1980–2007 ↑	2.65	0.51	2.78E−03	2007–2021 ↑↑	**–**	**–**	**–**	**–**
T20	0.88	0.88	2.84E−13	1980–2007 ↑	3.14	0.60	6.49E−04	2007–2021 ↑↑	**–**		**–**	**–**
T25	0.70	0.79	2.70E−10	1980–2007 ↑	2.38	0.79	9.49E−06	2007–2021 ↑↑	**–**	**–**	**–**	**–**
T30	0.54	0.82	4.29E−11	1980–2007 ↑	2.51	0.82	3.44E−06	2007–2021 ↑↑		**–**	**–**	**–**
T35	0.47	0.76	1.83E−09	1980–2007 ↑	2.45	0.76	2.17E−05	2007–2021 ↑↑	**–**	**–**	**–**	**–**
T40	1.86	0.91	8.33E−12	2000–2021 ↑	**–**	**–**	**–**	**–**	**–**	**–**	**–**	**–**
T45	1.63	0.91	5.39E−12	2000–2021 ↑	**–**	**–**	**–**	**–**	**–**	**–**	**–**	**–**
T50	1.40	0.81	1.09E−08	2000–2021 ↑	**–**	**–**	**–**	**–**	**–**	**–**	**–**	**–**
T75	0.98	0.75	2.09E−07	2000–2021 ↑	**–**	**–**	**–**	**–**	**–**	**–**	**–**	**–**
T100	0.83	0.85	7.91E−10	2000–2021 ↑	**–**	**–**	**–**	**–**	**–**	**–**	**–**	**–**
T500	0.09	0.51	1.87E−04	2000–2021 ↑	**–**	**–**	**–**	**–**	**–**	–	–	–

The letter “T” in abbreviations “T0, T1, etc.” means “threshold” that is applied to FPE values (see ranges in first column of Table 1). The dependency of the number of new genes in the respective FPE range as a function of the year is analyzed with linear regression methods. We omitted 2022 from this analysis because of the extremely small numbers, an artifact that might be caused due to COVID-19 restrictions. The trend of changes is generally identified through three phases, *i. e.* Phase 1, Phase 2 and Phase 3. The slopes, R^2^ and *P*-value in time intervals are listed based on linear regression model *y*_*i*_ ~ *C* + *b.x*_*i*_; where *y*_*i*_ is the total number of new genes reaching the specific FPE threshold at year *i*; *x*_*i*_ is the year *i*; *b* is the slope and *C* is intercept. A dash “- “ denotes not enough data. The slope (*b*) indicates the rate increase/decrease of the total number of new genes reaching a specific FPE score threshold throughout the years. A positive slope indicates that, as a trend, the total number of new genes reaching a specific FPE score threshold tends to be larger than in the previous year (or from year to year); a negative slope indicates otherwise. R^2^ is the square of correlation ρ or the goodness of fit of the linear regression. P-value is the statistical significance of the slope. The total number of genes reaching the specific FPE score threshold can then be estimated by: *N*_*i*_ ~ *N*_(*i−*1)_ + *y*_*i*_; where *N*_*i*_ and *N*_(*i−*1)_ are the total number of genes reaching the specific FPE score threshold at year *i* and (*i−*1) respectively

The accelerated growth of the number of genes in T10 and above during the period of decline for T0-T5 highlights the critical turning point in the research dedicated to the yeast genes’ functions. This dichotomy suggests a better risk-*versus*-success profile in research projects once the knowledge threshold associated with T10 has been achieved. This observation raises the question of how many articles are needed for the transition of a gene towards T10 status (see Additional file 3: File 4 for data). In Table 3, we show the actual number of research articles that various genes required to achieve a certain FPE threshold. The border between T5 and T10 genes is at ~ 30 articles. Thus, the floodgates for a dramatic increase of the gene’s literature corpus will be opened once about 30 articles have been published on it.

**Table 3:** The real number of scientific articles necessary to generate a literature body of given FPE intervals about a *S. cerevisiae* gene

Tindex	Min	Max	Median	Mean	SD
T1	1	49	4	5.52	5.22
T5	5	104	20	24.94	16.35
T10	10	317	42	47.72	27.92
T15	16	589	62	70.20	39.69
T20	22	897	82	92.48	53.33
T25	28	1168	102.5	114.50	69.50
T30	34	1514	122	134.51	87.02
T35	39	1165	146	154.03	77.88
T40	44	1387	167	176.80	91.91
T45	50	1594	186.5	196.53	104.07
T50	56	1821	199	216.56	119.63
T75	85	834	304	318.19	130.12
T100	116	1254	391	432.92	197.87
T500	1214	2941	2200	2164.86	619.38

The letter “T” in abbreviations “T0, T1, etc.” stands for “threshold” applied to FPE values (compare with Table 2). We list the minimal (Min), maximal (Max), median and mean (together with the respective standard deviation—SD) numbers of articles associated with genes in the year when they crossed certain literature thresholds. As a trend, the number of actual articles is 2–5 times larger than the FPE value itself

Table 4 shows how many years genes in various FPE threshold brackets needed to reach this publication status. Clearly, the general level of available research technology is of significance here. For genes that reached T0 after 2000 (wide availability of omics technologies) or after 1989 (routine gene sequencing is established), higher T-thresholds (> T75) were reached ~ 15 years earlier than for genes that had their T0 event in/after 1965 or even before.

**Table 4:** Years necessary to generate a literature body of given FPE intervals about a *S. cerevisiae* gene

Tindex	All Genes					Genes with T0 in year 1965 and later				
	Min	Max	Mean	Median	SD	Min	Max	Mean	Median	SD
T1	0	47	6.78	5	6.76	0	47	6.75	5	6.69
T5	1	50	15.78	15	8.32	1	50	15.70	15	8.18
T10	2	66	19.78	19	8.95	2	54	19.63	19	8.64
T15	4	73	22.01	21	9.26	4	52	21.79	21	8.75
T20	5	79	23.58	22	9.49	5	49	23.26	22	8.70
T25	6	83	24.54	23	9.78	6	51	24.10	23	8.61
T30	7	85	25.81	24.5	10.40	7	54	25.29	24	8.96
T35	8	88	26.67	25	10.60	8	53	26.04	25	8.80
T40	8	88	27.17	26	10.37	8	53	26.53	26	8.52
T45	9	89	27.91	27	10.59	9	54	27.23	27	8.59
T50	9	88	28.29	28	10.54	9	55	27.66	27	8.70
T75	12	87	29.57	30	9.32	12	52	29.28	29	8.53
T100	14	89	30.79	30	9.12	14	55	30.36	30	7.91
T500	28	45	36.57	36	4.85	28	45	36.57	36	4.85

Tindex	Genes with T0 in year 1989 and later					Genes with T0 in year 2000 and later				
	Min	Max	Mean	Median	SD	Min	Max	Mean	Median	SD
T1	0	30	6.45	5	5.99	0	22	6.75	6	5.05
T5	1	32	14.25	14	6.32	1	22	12.53	13	4.34
T10	3	32	17.28	18	6.01	3	22	14.19	14	4.20
T15	4	33	18.89	19	5.96	5	22	14.29	14.5	4.14
T20	5	32	19.94	20	5.84	6	22	13.93	14	3.80
T25	6	33	20.52	21	5.53	7	21	14.85	15	3.60
T30	7	33	20.97	21.5	5.47	8	22	15.26	15	3.65
T35	8	33	21.44	22	5.43	8	21	15.70	16	3.82
T40	9	33	21.90	22	5.42	9	21	16.36	17	3.91
T45	10	33	22.24	23	5.53	10	21	15.60	16	3.60
T50	10	33	21.90	22	5.31	10	21	15.92	15	4.07
T75	13	33	22.13	21	4.75	13	18	16.00	17	2.24
T100	15	32	23.55	23	4.44	15	21	17.25	16.5	2.63
T500	–	–	–	–	–	–	–	–	–	–

The letter “T” in abbreviations “T0, T1, etc.” stands for “threshold” applied to FPE values (compare with Table 2). We list the minimal (Min), maximal (Max), median and mean (together with the respective standard deviation—SD) numbers of years needed to accumulate the necessary FPEs for a given gene relative to the gene’s year for T0. As the research technology has dramatically improved compared with the time when the first genes achieved T0 (the first relevant recorded publication PMID:19872702 about invertase is from 1932), we also give the data for all eligible yeast genes as well as separately for those with their T0 event beginning with 1965 (molecular biology got established), with 1989 (gene sequencing became routine) and after 2000 (omics technologies got widely available). Notably, the years necessary for getting into higher T ranges tend to get smaller for more recently studied genes but still remain well above a decade (~ 15 years). Notably, the median number of years needed to make a threshold dropped more dramatically for higher literature thresholds (> 15 years for T75, T100 and T500 versus a drop by just 5–10 years for medium T-thresholds). The data emphasizes that, indeed, technological developments such as the introduction of omics technologies had a positive effect on the progress in gene function discovery

Genes that have been identified from year 1965 onwards needed approximately 20 years to achieve the T10 threshold, whereas for genes that have occurred in the literature only after the year 2000 onwards, it still takes approximately 14 years to achieve the same T10 threshold. It is interesting to note that the average number of years to achieve even much higher FPE thresholds for genes first mentioned in year 2000 onwards is only a few years (~ 3 years) longer than the time to achieve the T10 threshold, which further suggests that T10 appears be the threshold critical to overcome scientific risks and technical problems and to trigger more incremental type of research for those yeast genes.

The only exception from the general speed-up in research progress appears to be T1. The mean number of years to reach level T1 after T0 has been achieved remains essentially constant (between 6.45 and 6.78). Thus, the technology progress has not much effect at this level of gene function understanding and, for each gene, some ingenious ideas (the critical hindrance) seem to be required.

**Fig. 2:**
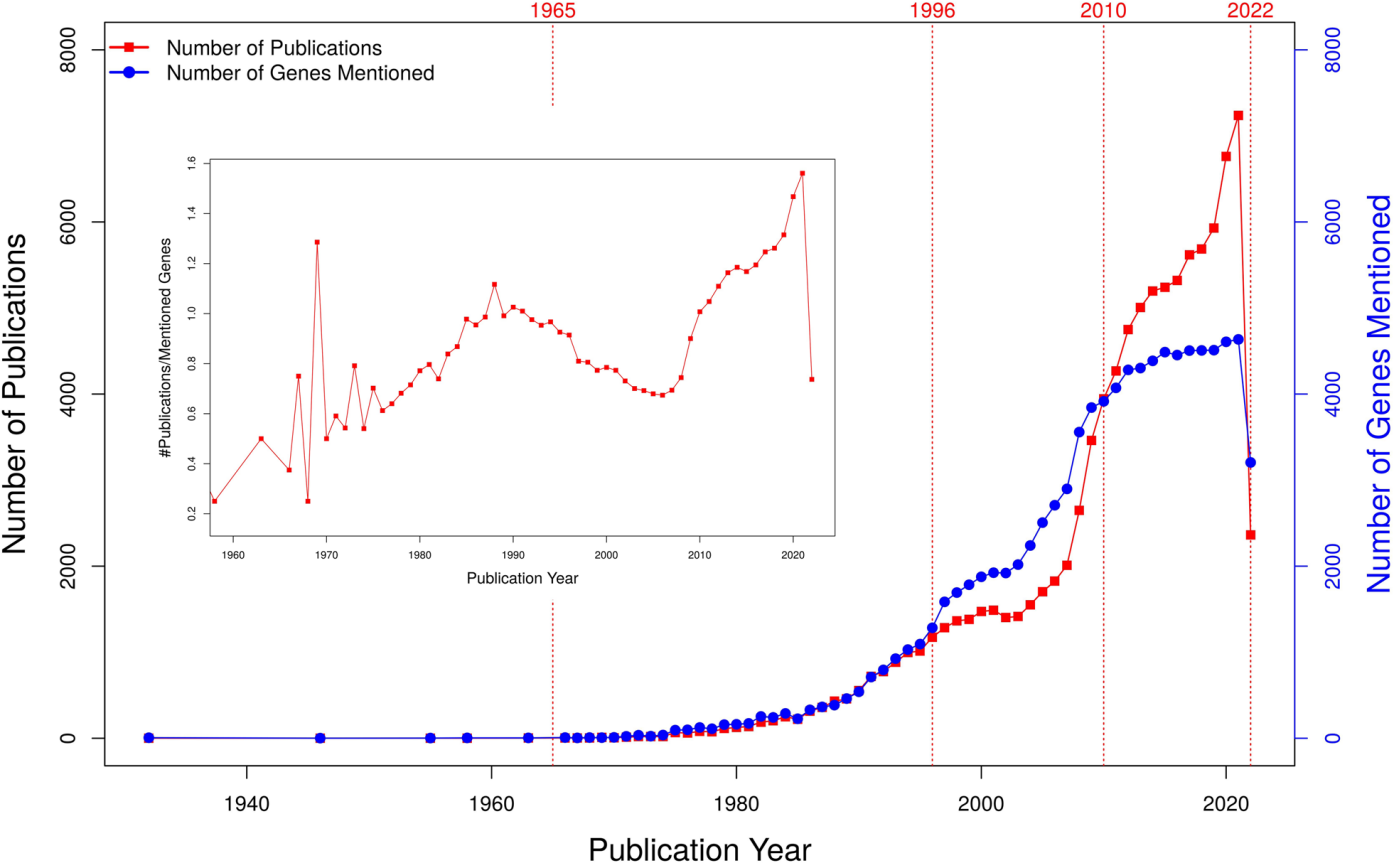
The number of mentioned genes per year in relation to the total number publications with Yeast genes from year 1932–2022. We show the dynamics of the yeast gene function-related publications in connection with the number of genes mentioned per year. The number of publications (left y-axis) for each year is represented by the red line, whereas the number of genes mentioned per year (right y-axis) is shown by the blue line. Publication about yeast genes started to become frequent beginning with the year 1965. After 1996, we observed an increase in the number of genes mentioned per year, which coincides with the release of the first genome sequence of yeast. This phenomenon saturated around year 2010 where the number of publications keeps growing while the number of genes mentioned have plateaued

These data should be seen at the background of the total literature corpus published about yeast genes over the years (Fig. 2). The number of new relevant publications (red curve) knows only an upwards trend over the years (except for 2022, most likely a delayed effect of the work-from-home policies during COVID-19 that prevented laboratory experimentation). Clearly, there has been a saturation in the number of genes (blue curve) mentioned in any article of that year since ~ 2007 and a steep increase of the number of articles per mentioned gene since the same year. Thus, any decline in gene function discovery is not due to an overall decrease of scientific articles published.

### Contribution of various scientific journals to the yeast gene function discovery

As a side effect of our literature survey, each fractional count for a given gene and a given journal article can be associated with any of the 15 FPE threshold qualifiers T0, T1, …, and T500. If summed up for a given journal, we can calculate how many T0-, T1-, …, T100-, or T500-type publications a journal has accumulated over certain historical periods. Thus, each journal can be characterized by a 15-dimensional vector with FPE values corresponding to the T-thresholds (Additional file 3: File 5).

We analysed the spatial association of the journals in the 15-dimensional T-space with principal component analysis. We find that ~ 90% of the data variation is explained by the first principal component, ~ 8% by the second, ~ 1% by the third. The top loadings for the first principal component come from T10 to T75 (correlation coefficients > 0.95 with any of these coordinates; Additional file 1: Table S1). The second principal component is largely influenced by T0, T1, T100 and T500 and correlates with the T0-T500 difference (correlation coefficient ~ 0.98; Additional file 1: Table S1).

**Fig. 3:**
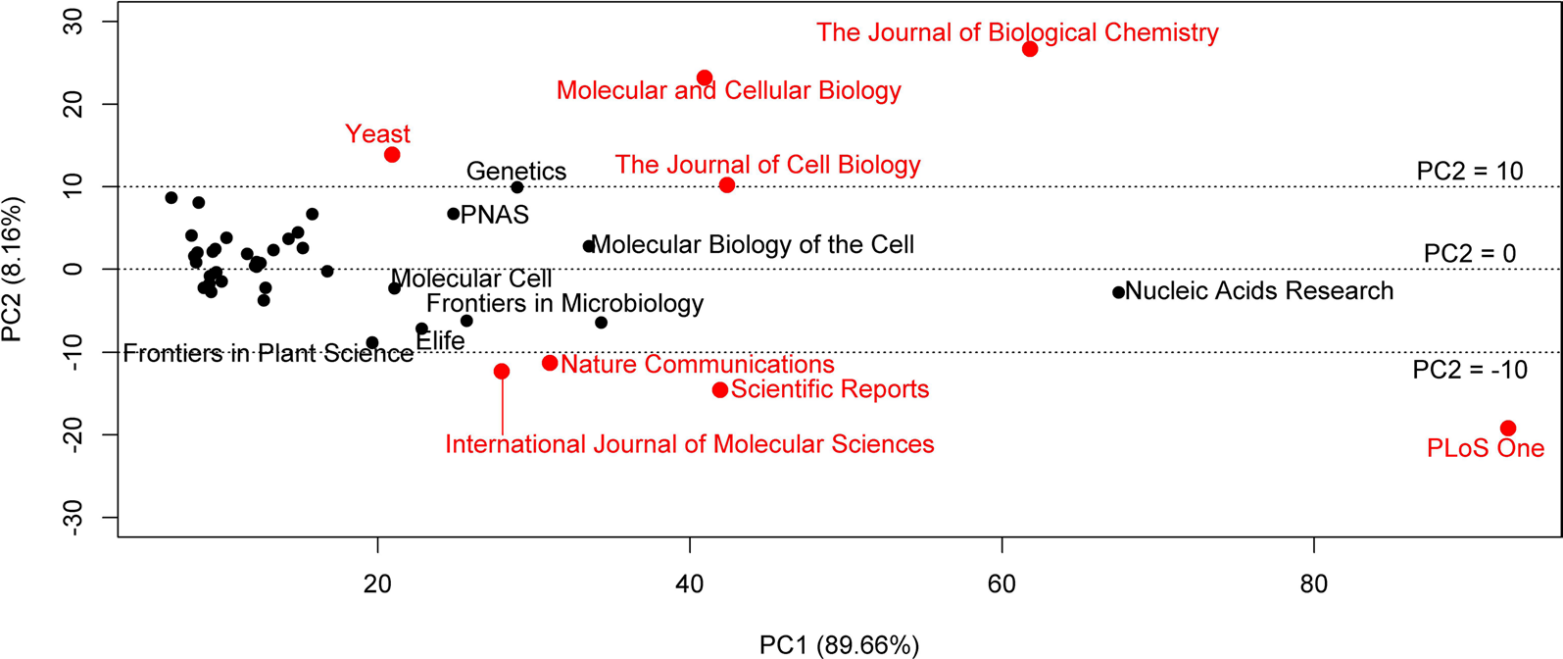
Contribution of various scientific journals to the literature about functions of *S. cerevisiae* genes. The journals in our text corpus that contribute to the literature about baker’s yeast gene functions (minimum 500 scientific articles) can be characterized by a 15-dimensional vector of T-thresholds with the respective aggregated FPEs (T1, T5, T10, …, T50, T75, T100 and T500). We show these vectors in their projections onto the plane of the first (PC1, x-axis) and second (PC2, y-axis) principal components (principal component analysis for all journals found). The first principal component reflects the total number of papers about yeast biomolecular mechanisms published by the journal. The second principal component shows whether the journal is strong in publishing early (T0 or T1; with large positive PC2) papers or late (T100 or T500; with negative PC2 having large absolute value) papers about yeast genes

The relevant journals in our text corpus are shown in their projections onto the plane of the first and second principal components (Fig. 3, Additional file 3: File 5). Whereas the first principal component is rather affected by the total number of papers about yeast biomolecular mechanisms, the second principal component shows whether the journal has an edge in publishing early (T0 or T1) papers or late (T100 or T500) papers about yeast genes.

A few journals are clearly outliers compared to the crowd (outside the bracket [− 10, 10] for PC2). We find that “The Journal of Biological Chemistry” (with 8.9% of all T0 and 7.2% of all T1 yeast gene papers in history), “Molecular and Cellular Biology” (with 8% of all T0 and 5.3% of all T1 papers), “The Journal of Cell Biology” (with 5.7% of all T0 and 3.7% of all T1 papers)) and “Yeast” (with 5.7% of all T0 and 2.6% of all T1 papers) are the forerunners of publishing T0 + T1 papers about yeast genes. The journals “PLOS One”, “Scientific Reports”, “Nature Communications” and “International Journal of Molecular Science” are comparatively strong with T500 and T100 publications.

For each of the eight selected journals, Additional file 2: Fig. 3A–H shows the components of the T-threshold vector. Here, the y-axis value for a given T-threshold is the fraction of all T-threshold publications of the respective journal from the total pool of the same T-threshold publications in any journal (calculated in terms of FPEs). We also show a regression line as indicator of the trend along the T-threshold (Tindex) vectors together with the slope and the significance. The qualitative difference between the two groups of journals is visually striking. Whereas the first four journals show a clear, significant decline towards T500, the four remaining ones exhibit a convincing rise towards higher T-thresholds.

A thorough investigation of the connection between the journal’s impact factor (IF) and the journals’ role in publishing early research about genes’ function is beyond the scope of this work. Yet, a quick analysis for the most extremely positioned journals in Fig. 3 shows that there might be some trend.

The IFs (2-year impact) of the journals belonging to the first group are 5.48, 8.08, 5.09 and 3.32, respectively (taken from [14] at the time of writing; the average is 5.49). Those for the second group are 17.00, 5.00, 3.75, and 6.21 (average 8.16). Obviously, the IFs in the latter group tend to be higher (yet the T-test is non-significant). Apparently, there is some trend that journals with higher IF are rather part of the second group of journals. At the same time, avoiding publishing early gene function discovery papers does not guarantee a high impact IF.

## Discussion

Funding for early full genome sequencing of model organisms was justified with the argument that knowledge of the whole genome sequence would enable systemic approaches towards the network of pathways and gene networks due to the completeness of the gene list [4]. Clearly, this requires the availability of the list of all gene functions including their hierarchical description with all molecular, cellular and phenotypic functional aspects (see Fig. 1 in [15]). Few would have guessed that, even many decades later, the yeast gene function list remains largely incomplete. Even more, the thrust of the scientific community to solve these problems seems to be diminishing rather than gaining momentum.

Neither with the skew in the literature towards a few elite genes nor with the decline of new gene function discovery, yeast is a special organism. The situation is similar for other model organism such as *E. coli* [10] or human [9, 16, 17].

It is especially worth noting that the decline of new gene function discovery happens not at the background of a general decline of yeast research. As shown in Fig. 2, the academic research machine continues humming and churns out an ever-increasing number of papers on biomolecular mechanisms involving yeast genes.

Our data allow us to estimate the time needed to achieve a complete yeast protein-coding gene function list under the assumption that the status from the recent past can be extrapolated into the future. During the past few years, the number of newly appearing yeast gene names (T0 articles) in the scientific literature was in the range ~ 30 (Additional file 3: File 3). If this value does not decrease further (an apparently optimistic assumption given the trend during the last ~ 25 years), it will take another > 20 years (until ~ 2050?) before every yeast gene has a least a single literature mentioning besides any occurrences in data sheets from high-throughput omics studies, genetic/mutation screens, or large-scale subcellular localization assays.

Further, the threshold T5 (5 FPEs correspond to ~ 20 publications involving the gene (see Table 3 and refs. [9, 10]), a somehow reasonable status of research success) is crossed by ~ 70 yeast genes every year in the more recent history. With currently almost 4000 genes below T5, it will require another ~ 55 years to reach a decent level of function description for all yeast genes; thus, we can speak about ~ 2080 until the last remainders are covered. T10 is crossed for ~ 100 genes per year. Therefore, it will take ~ 50 years more from today for the ~ 4800 genes to get there.

Pena-Castillo and Hughes [5] tried to investigate several possible causes that might delay yeast gene function discovery. Among the questions, they considered:

1. Are uncharacterized genes real?
2. Are the uncharacterized genes too new to have been studied?
3. Do uncharacterized genes have any distinguishing characteristics in large-scale analyses?
4. Are the uncharacterized genes needed only under specific conditions that are not easily available during standard laboratory experiments?

The authors conclude that, most likely, the overwhelming number of uncharacterized yeast genes is real though the identification of genes coding small [18] and orphan [19] proteins is difficult. The investigations of uncharacterized genes are hampered by functional redundancy, lack of strong phenotype or even absence of expression in standard laboratory experiments. Yet, the advance in research technology, especially of omics approaches and bioinformatics, should and does give hints that eventually should lead to function discovery [20].

Our data in Table 4 indeed factually supports this insight. Omics studies can broadly assign uncharacterized genes to processes and phenotypes and, thus, direct follow-up research. For example in the work of Wood et al. [21], the authors identify groups of conserved but still unstudied proteins in *Saccharomyces pombe* (fission yeast) based on a combination of large-scale experimental data and bioinformatics analyses. Ingenuity and enthusiasm of researchers today is certainly not smaller than several decades years ago when people struggled with truly primitive research tools as we understand it now [20]. There are efforts to launch research initiatives to discover functions of unstudied genes based on omics findings [22, 23].

Nevertheless, yeast gene function discovery has come out of fashion when many hundreds of yeast genes still require research attention. The fact that the decline of new function discovery happens at the background of an expanding academic research capacity certified by the continuously growing number of relevant publications indicates that other, not intrinsically scientific factors have a role in the change. Similar trends have been observed in many other areas of science [24–26]. Besides some lamenting, the signs of decline have been rather considered a natural phenomenon than a man-made societal change [27]. Polite comments in previous publications hint towards intrinsic transformations that have distorted the academic system in the past five decades and have driven it increasingly ineffective [9, 10, 28, 29].

Our data shows that new gene function discovery is a process that occupies ~ 15 years or more (arrival at T10 or better) after the first publication has appeared. Almost half of this time (~ 7 years) is required for the gene just to get from label T0 to T1. Once the T10 threshold is reached, a body of ~ 30 papers is created with associated research costs not below USD 8 million.

If these numbers are compared with typical contractual conditions of young faculty members and their grants (time slots typically ≤ 5 years and grant sizes per PI in the order of a few USD 100,000 at best), it becomes clear that they expose themselves to great existential risk if they start working on genes that really attracted no attention before.

Maintaining a research team financially is a difficult task for a young principal investigator and academic research grants are the main source. For those in the know, getting funding for research on uncharacterized genes just for the purpose of finding the function is very difficult if only for the reason of absence of preliminary data or the unclear future application of the result.

The evaluation of journal publications with publicity metrics (such as IFs that in reality measure the size of the audience and the time of reaction on the publication of the original article) further complicates the path for gene function discovery. Our data does not provide any evidence that publishing first papers about a gene adds to the IF of a journal. Thus, the pressure for a high IF paper also drives researchers away from working on uncharacterized genes as, at least at the beginning, fewer people will be interested in their work, and it will take usually more than two years for any follow-up paper to cite them. The more laudable are those journals who jump in to support the early results reaching the community.

Did the full genome sequencing of yeast boost gene function discovery? Unfortunately, the promises that justified the investment for yeast’s genome sequencing did not materialize. The data proves not only that there was no boost from the public availability of all gene sequences. Tragically, the moment of publishing the full genome sequence in reality coincides with the onset of decline of gene function discovery for previously uncharacterized genes. Even more disappointing is the insight that, if the current trends for the literature about yeast molecular mechanisms can be extrapolated into the future, it will take about another 50 years to complete the yeast gene function list.

## Methods

Technically, this work is similar to previously published research [9, 10]. In brief, we reused the text corpus, gene name dictionaries and the mapping procedure from version 11.5 of the STRING database [12, 13]. Issues of accuracy of the automated assignment procedure are discussed in detail in our previous work. To note, for the suppression of false-positive assignments, we apply an explicit rule system, based on regular expressions and a list of blocked names [11], to suppress the detection of entity names in target texts when the respective words are frequently used have another, normal English meaning. These resources are continuously updated. We used the release labelled with the 19th of June 2023.

In accordance with previous work, we rely on fractional counting of entity names and sum them up for the determination of full publication equivalents (FPE). Typically, a text document mentions multiple genes/proteins. Each paper that mentions at least one gene/protein contributes an FPE of 1, which is spread across the mentioned gene/proteins depending how many times each of them was mentioned. Thus, the total fractional count *f*_*i*_ for protein or gene *i* is

$$ f_{i} = \mathop \sum \limits_{j \in D} \frac{{n_{ij} }}{n \cdot j} $$

Here, *D* is the document set, ${n}_{ij}$ is the number of times protein or gene *i* is mentioned in document *j*, ${n}_{\bullet j}$ is total number of mentions of any gene/protein in document *j*.

In our master file (Additional file 3: File 1), each line contains a genomic entity name, a publication identifier, the publication date, and the fractional count associated with that genomic entity name. Herewith, it is straightforward to determine the amount of literature published about a given genomic entity in certain time periods by summing up the respective fractional counts.

The software “R” and Microsoft Excel were applied for data manipulation, principal component analysis, and further statistical tests.

### Supplementary Information

**Additional file 1. Table S1**. We show the correlation of T-threshold (Tindex) coordinates (T0, T1, …, T500) and coordinate differences (T0-T500 and T1-T500) to PC1 and PC2 based on the journals with at least 500 relevant articles.

**Additional file 2. Figure S1**. Explanation of variance of journals’ T-threshold data by principal coordinates. The graph illustrates how much of the data variance in the journals T-threshold vectors is explained by which principal coordinate. **Figure S2**. Loadings of principal coordinates PC1 and PC2. The loadings of principal coordinates PC1 and PC2 to the T-threshold coordinates (Tindex) are shown. **Figure S3**. Illustration of T-threshold vectors for selected journals together with indicative regression lines. The figures illustrate the T-threshold (Tindex) vectors for selected journals, the outliers in Figure 3 (four journals strong in T0, T1, … publications: “The Journal of Biological Chemistry”, “Molecular and Cellular Biology”, “The Journal of Cell Biology”, and four journals with overweight of T500, T100, T75, … publications: “PLOS One”, “Scientific Reports”, “Nature Communications” and “International Journal of Molecular Science”). Here, the y-axis value for a given T-threshold is the fraction of all T-threshold publications of the respective journal from the total pool of the same T-threshold publications in any journal (calculated in terms of FPEs). We also show a regression line as indicator of the trend along the T-threshold (Tindex) vectors together with the slope and the significance. Whereas the first four journals show a clear, significant decline towards T500, the four remaining ones exhibit a convincing rise towards higher T-thresholds. (A) The Journal of Biological Chemistry. (B) Molecular and Cellular Biology. (C) The Journal of Cell Biology. (D) Yeast. (E) PLOS One. (F) Scientific Reports. (G) Nature Communications. (H) International Journal of Molecular Science.

**Additional file 3. File 1**. This file lists the association between the gene and the article mentioning the gene (defined by the PUBMED ID). Count defines the number of times a gene is mentioned in the associated article. FPE is the FPE-score of the gene in the associated article. **File 2**. This file lists the yeast-relevant literature items with their publication year. **File 3**. This file lists the number of new genes reaching a specific FPE threshold as defined by T-threshold categories throughout the years. **File 4**. This file provides the information of which year the gene (GeneID) is first mentioned (First Mentioned). Subsequently, we provide the number of articles that have been published for the gene until it reaches a specific FPE threshold (T1, T5, T10, ..., T100, T500). The column with "−" value means the gene has not achieved that FPE threshold in the study till December 2022. **File 5**. This file provides the list of journals publishing the yeast relevant articles until December 2022. The total number of articles for each journal is listed. The projected principal components for the 15 T-threshold categories are given as PC1, PC2 until PC15. Subsequently, the percentage of the contributed FPE score for each journal is given for T0 through T500.

## Data Availability

All data generated and analyzed during this study are included in this published article and its supplementary information files. Additional file 1 provides tables. Additional file 2 contains figures. The zip-package Additional file 3 provides a legends file with content description of further files contained in the package.

## Abbreviations

FPE: Full publication equivalent
ID: Identifier
IF: Impact factor
ORF: Open reading frame
*S. cerevisiae*: *Saccharomyces cerevisiae*
