# Supplementary material for: Did the early full genome sequencing of yeast boost gene function discovery?
Source: Biol Direct. 2023 Aug 14;18:46. doi: 10.1186/s13062-023-00403-8 (PMC10424406; doi:10.1186/s13062-023-00403-8)
Supplement: Supplementary file 1 — Additional file 1. Table S1. We show the correlation of T-threshold (Tindex) coordinates (T0, T1, …, T500) and coordinate differences (T0-T500 and T1-T500) to PC1 and PC2 based on the journals with at least 500 relevant articles. [file 13062_2023_403_MOESM1_ESM.pdf]

Supplementary Table S1: Correlation of Tindex, (T0-T500) and (T1-T500) to PC1 and PC2 based on the journals with at least 500 articles

Tindex	PC1	PC2
T0	0.5228	0.7684
T1	0.7028	0.6254
T5	0.9041	0.3184
T10	0.9715	0.1173
T15	0.9883	0.0108
T20	0.9923	-0.0595
T25	0.9878	-0.1550
T30	0.9834	-0.1472
T35	0.9805	-0.1980
T40	0.9751	-0.2421
T45	0.9529	-0.3524
T50	0.9563	-0.3138
T75	0.9543	-0.3435
T100	0.8894	-0.5237
T500	0.7110	-0.6766
Diff (T0 – T500)	0.0108	0.9804
Diff (T1 – T500)	-0.0080	0.9693
