# Supplementary material for: Did the early full genome sequencing of yeast boost gene function discovery?
Source: Biol Direct. 2023 Aug 14;18:46. doi: 10.1186/s13062-023-00403-8 (PMC10424406; doi:10.1186/s13062-023-00403-8)

Supplementary Figure S1
Explanation of variance of journals' T-threshold data by principal coordinates

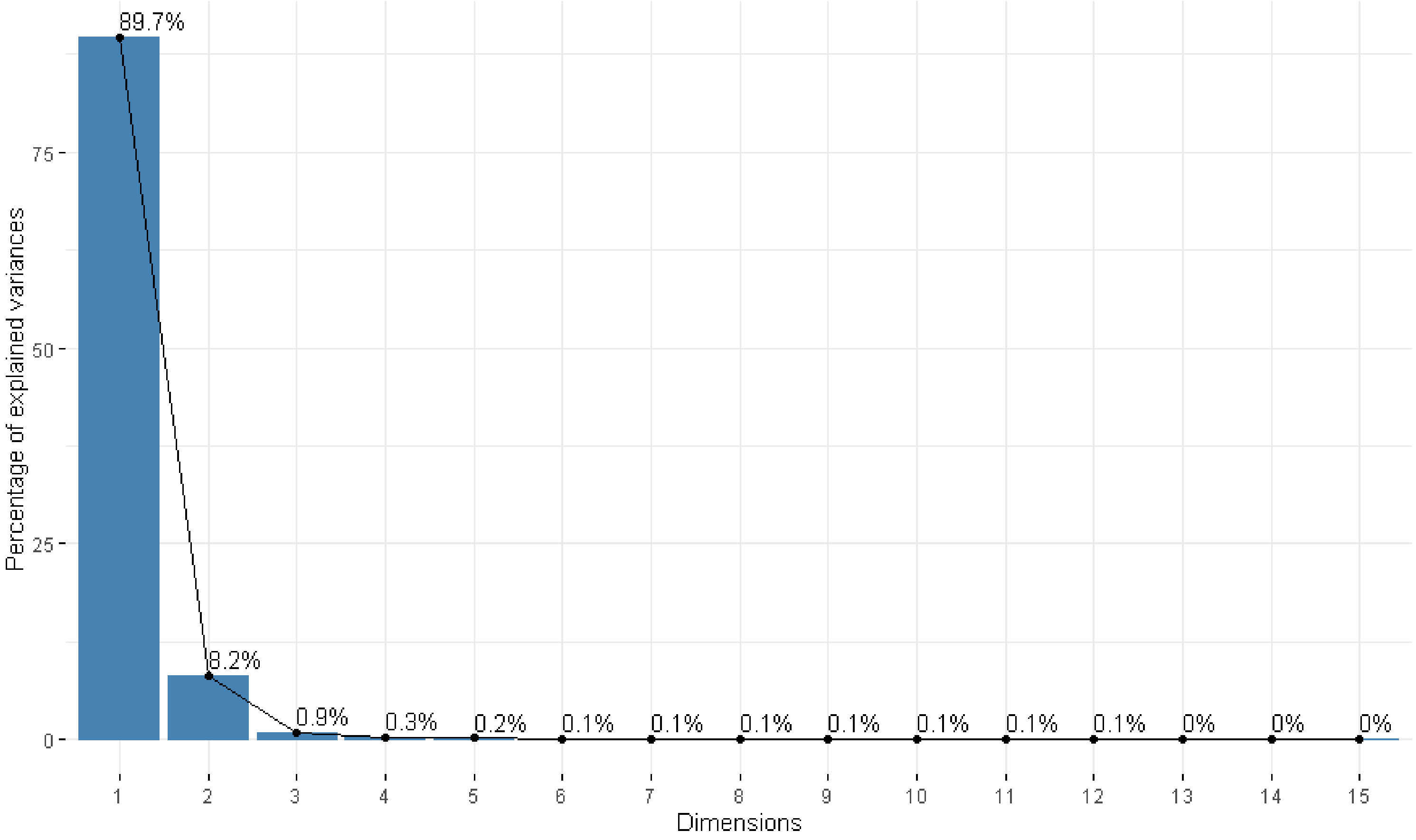

Supplementary Figure S2
Loadings of principal coordinates PC1 and PC2

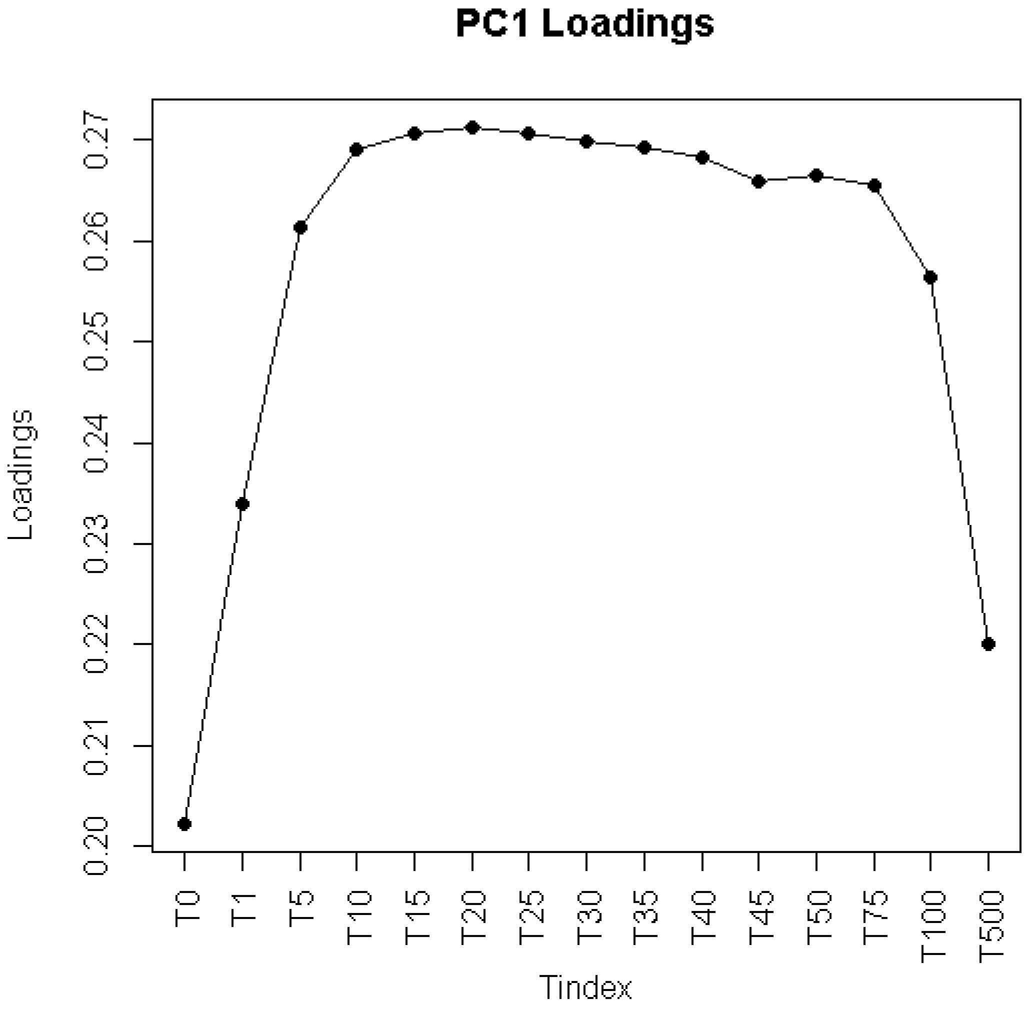

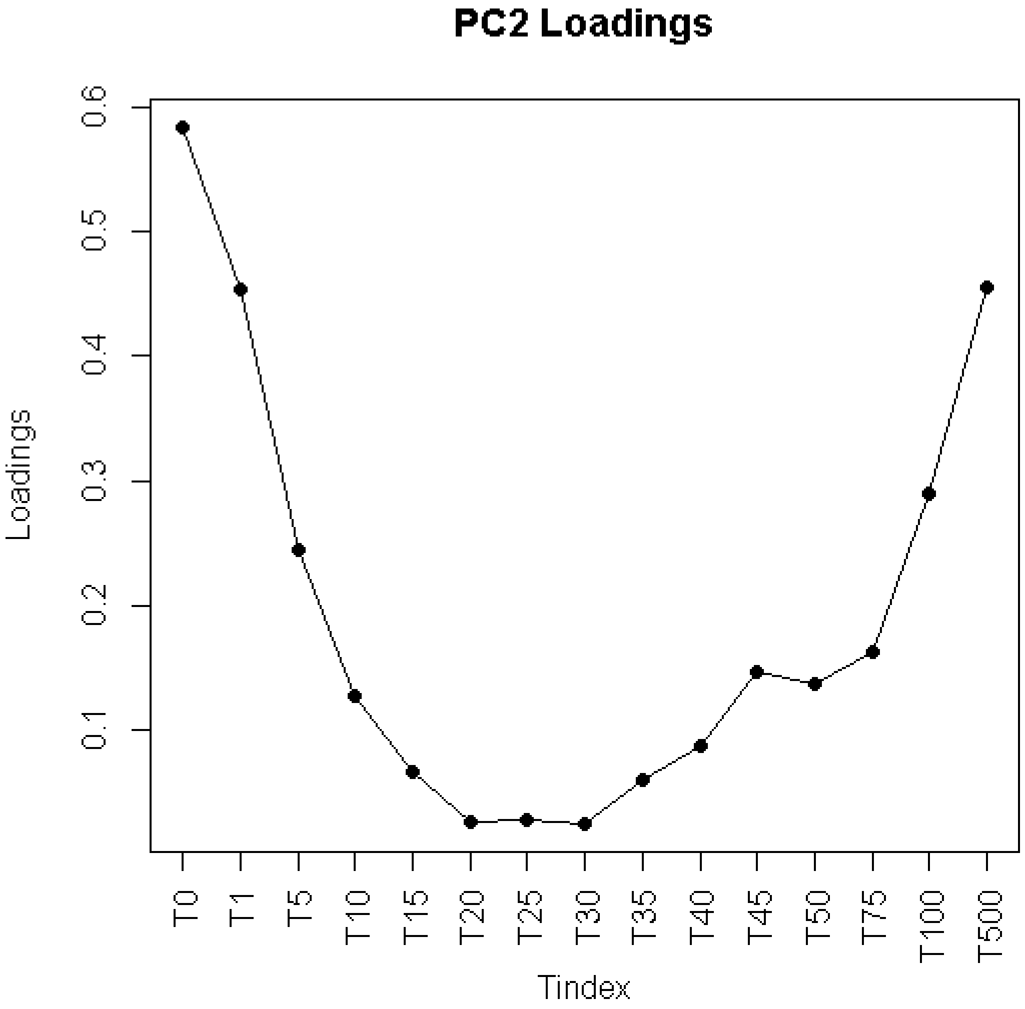

Supplementary Figure S3 – Illustration of T-threshold vectors for selected journals together with indicative regression lines

The figures illustrate the T-threshold (Tindex) vectors for selected journals, the outliers in Figure 3 (four journals strong in T0, T1, … publications: “The Journal of Biological Chemistry”, “Molecular and Cellular Biology”, “The Journal of Cell Biology”, and four journals with overweight of T500, T100, T75, … publications: “PLOS One”, “Scientific Reports”, “Nature Communications” and “International Journal of Molecular Science”).

Here, the y-axis value for a given T-threshold is the fraction of all T-threshold publications of the respective journal from the total pool of the same T-threshold publications in any journal (calculated in terms of FPEs). We also show a regression line as indicator of the trend along the T-threshold (Tindex) vectors together with the slope and the significance. To note, the regression results are just indicative for the trend since the T-threshold axes is not evenly linear (the metric between successive T-thresholds varies). Whereas the first four journals show a clear, significant decline towards T500, the four remaining ones exhibit a convincing rise towards higher T-thresholds.

A) The Journal of Biological Chemistry
B) Molecular and Cellular Biology
C) The Journal of Cell Biology
D) Yeast

E) PLOS One
F) Scientific Reports
G) Nature Communications
H) International Journal of Molecular Science

Supplementary Figure S3A

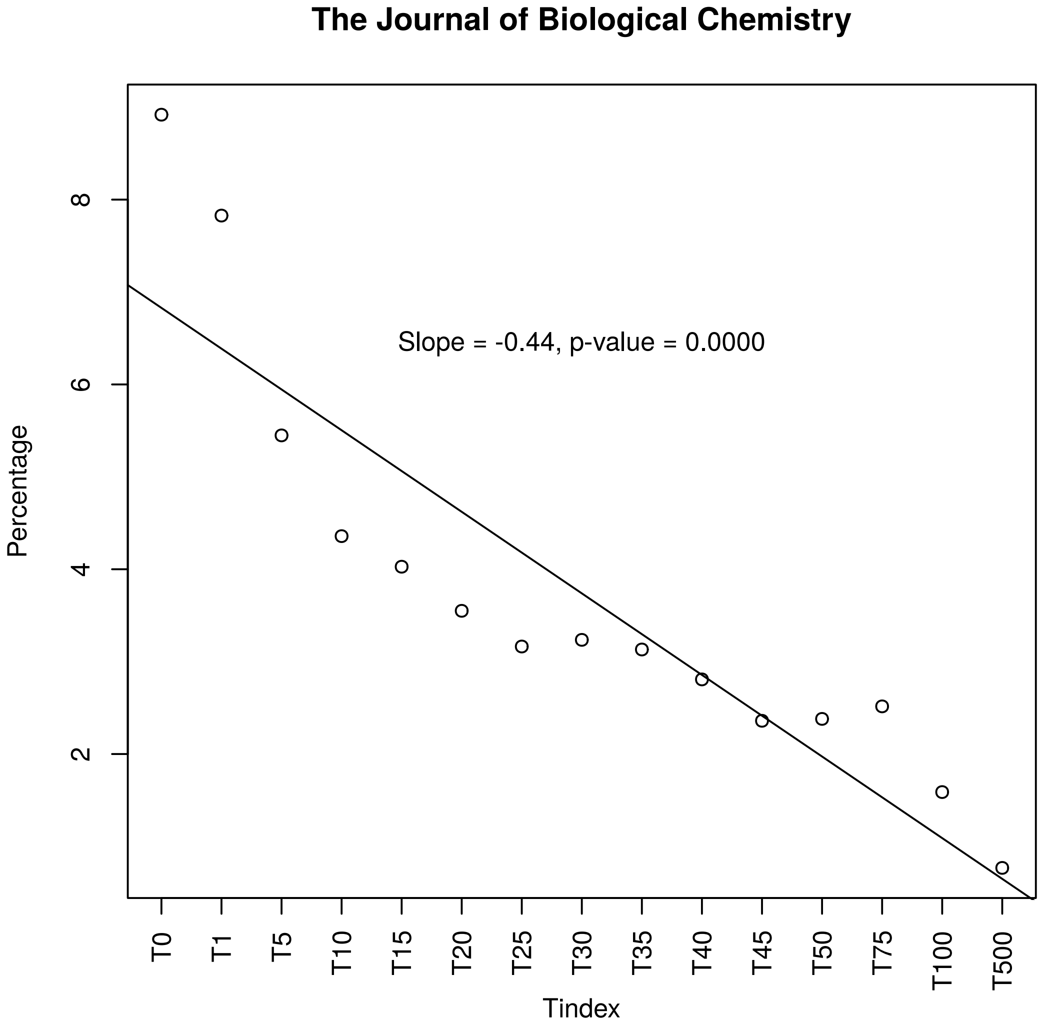

Supplementary Figure S3B

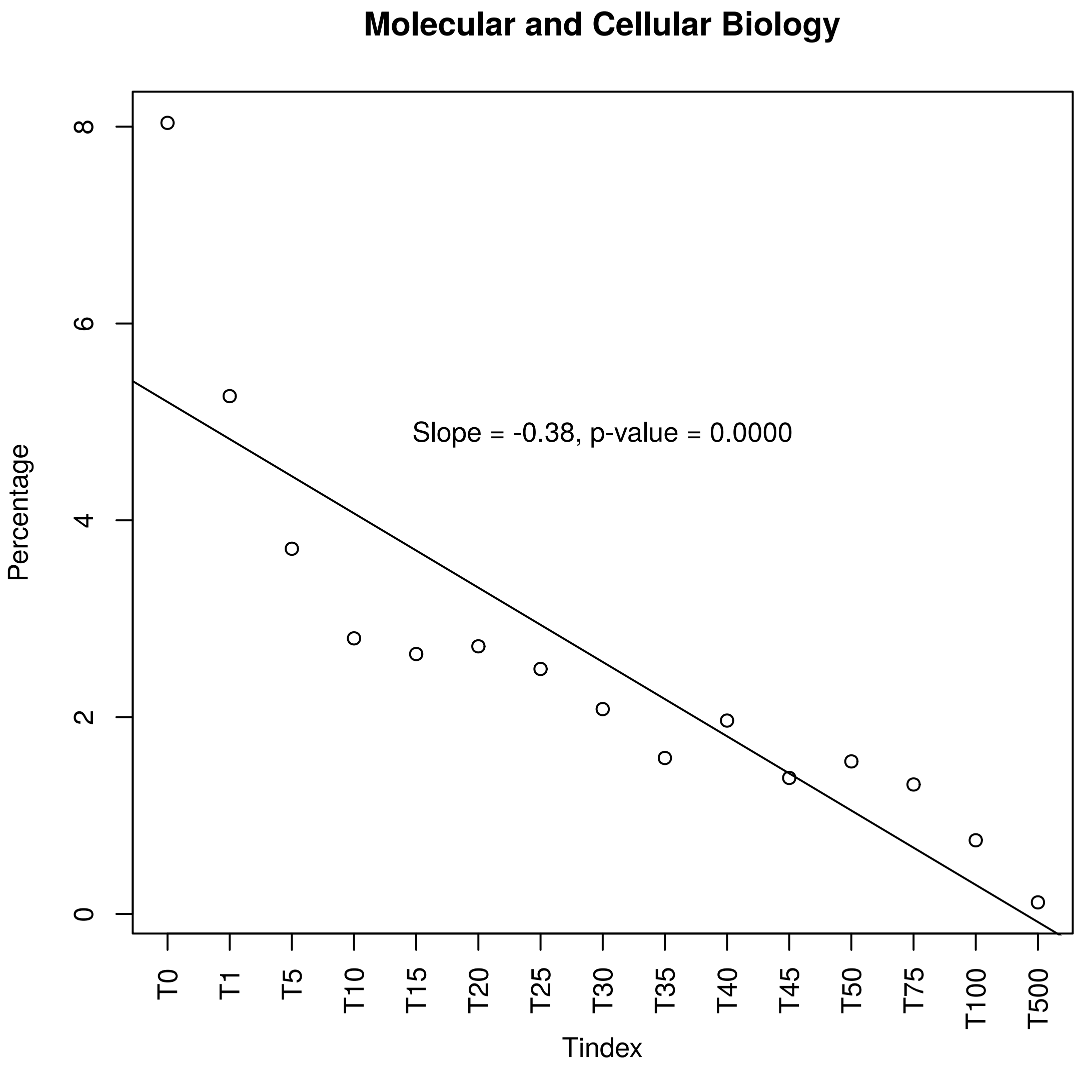

Supplementary Figure S3C

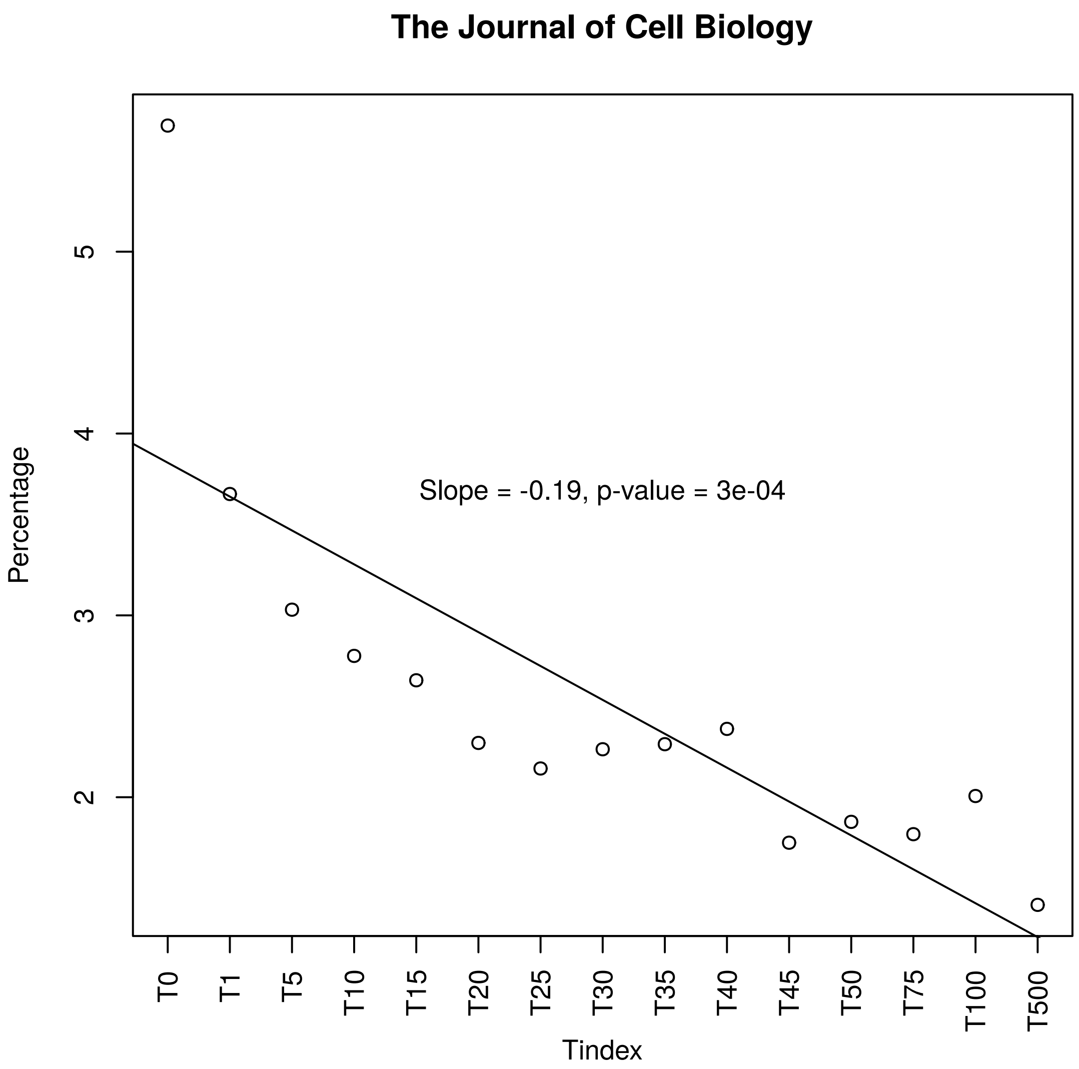

Supplementary Figure S3D

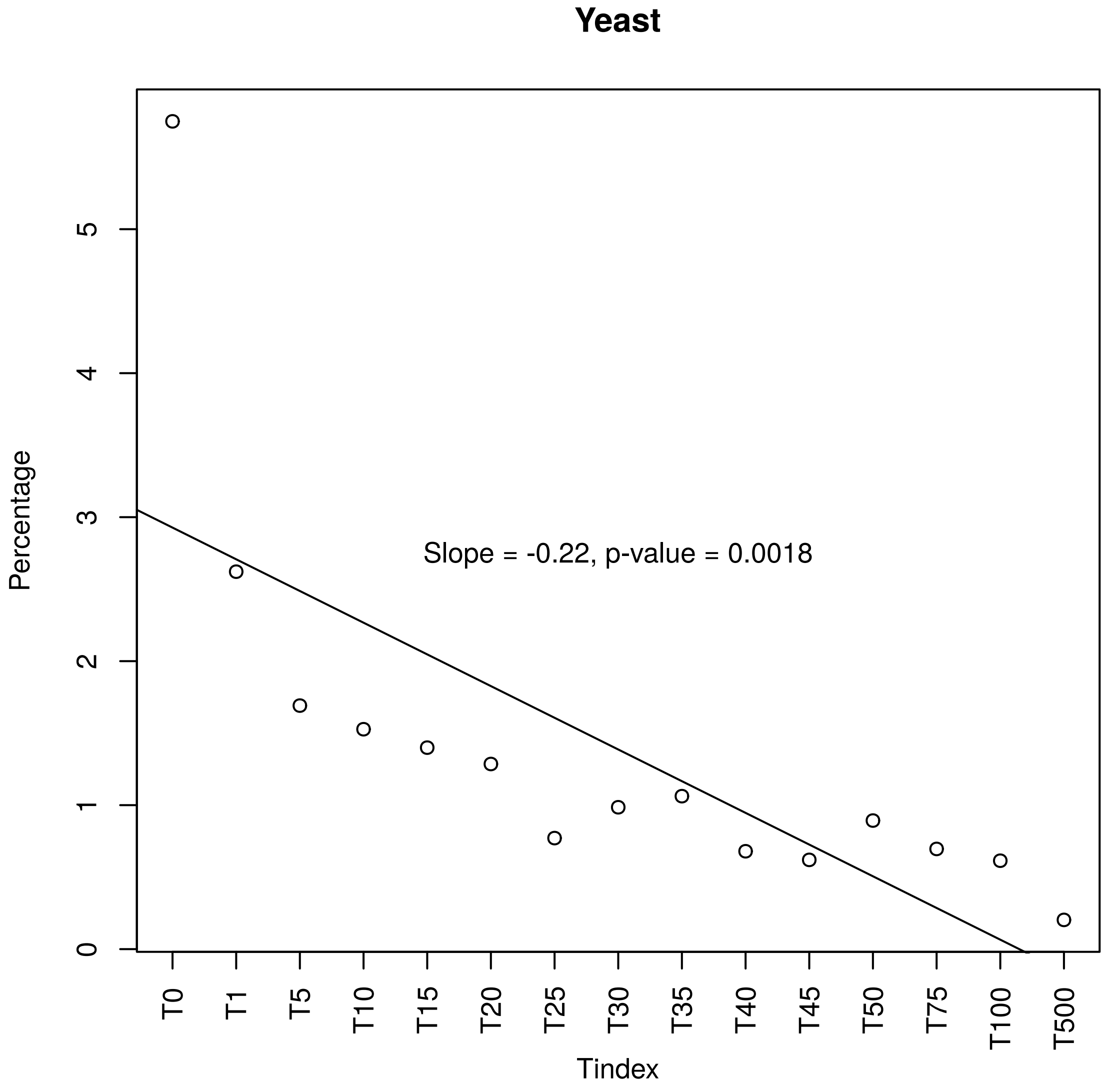

Supplementary Figure S3E

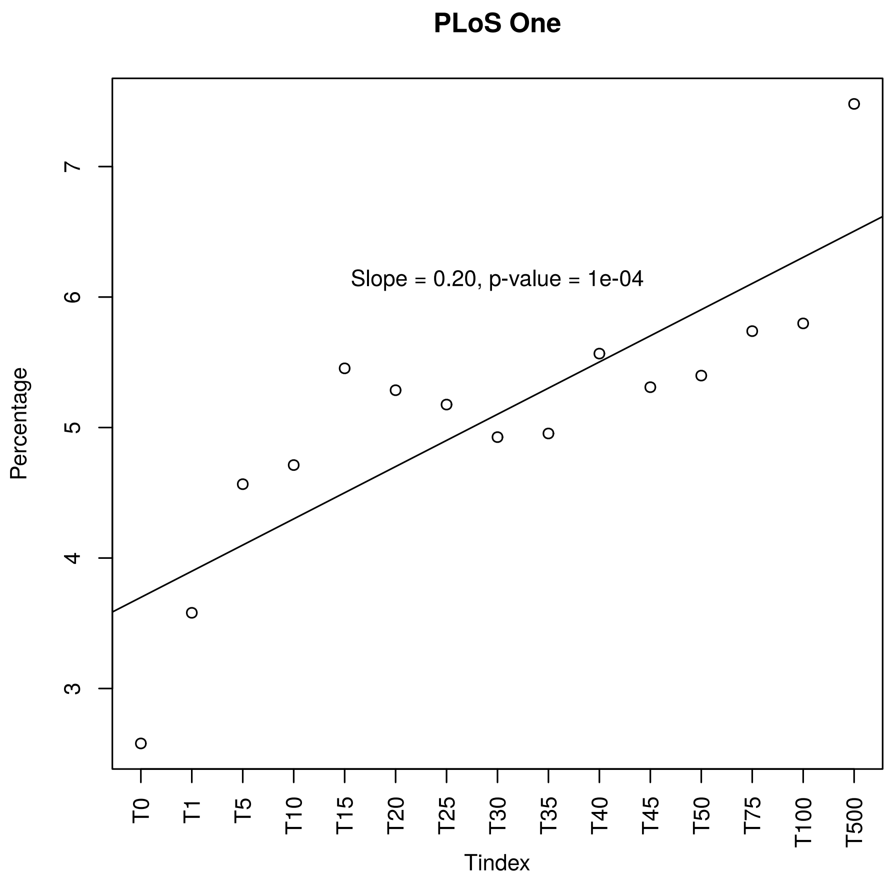

Supplementary Figure S3F

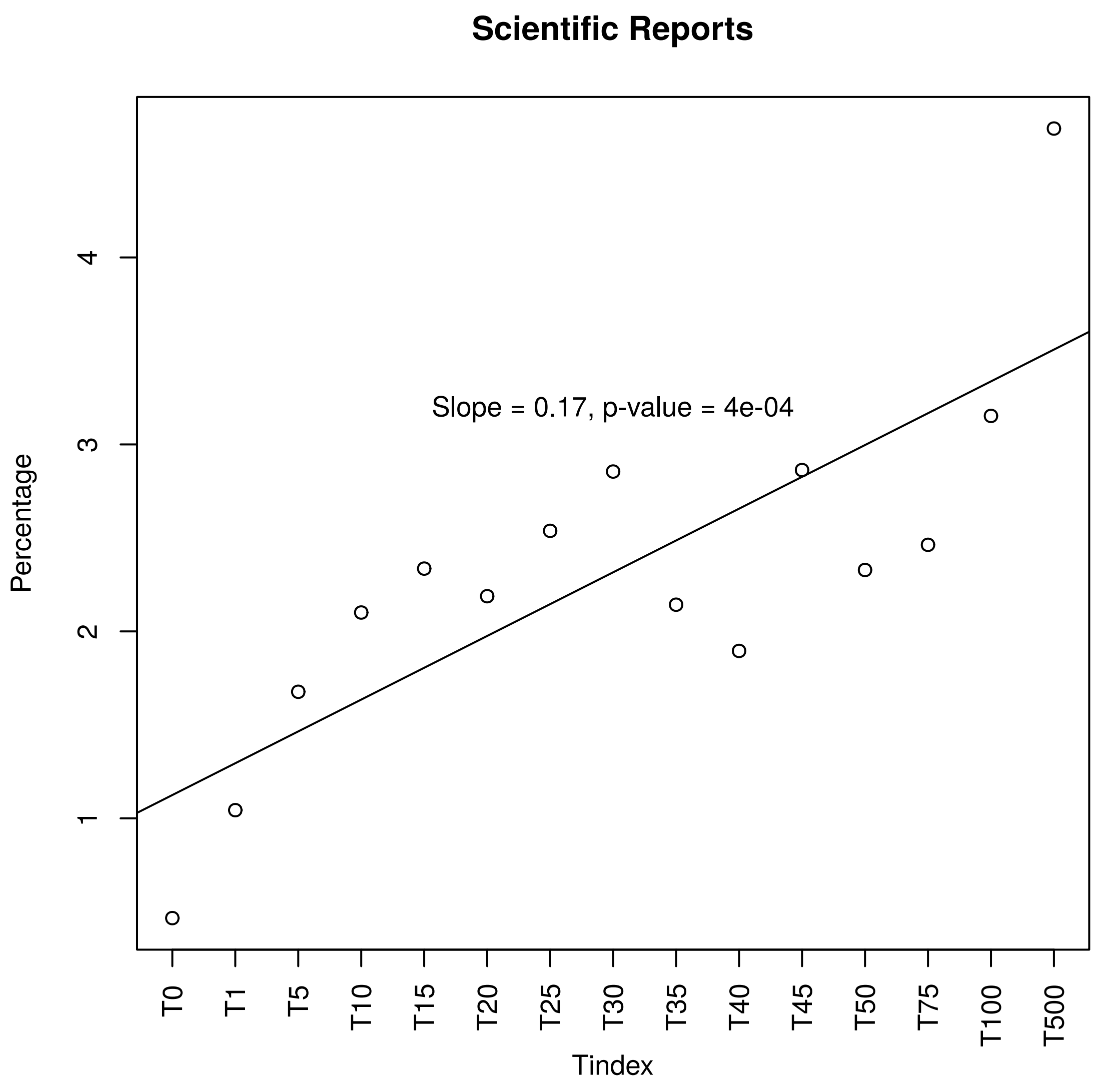

Supplementary Figure S3G

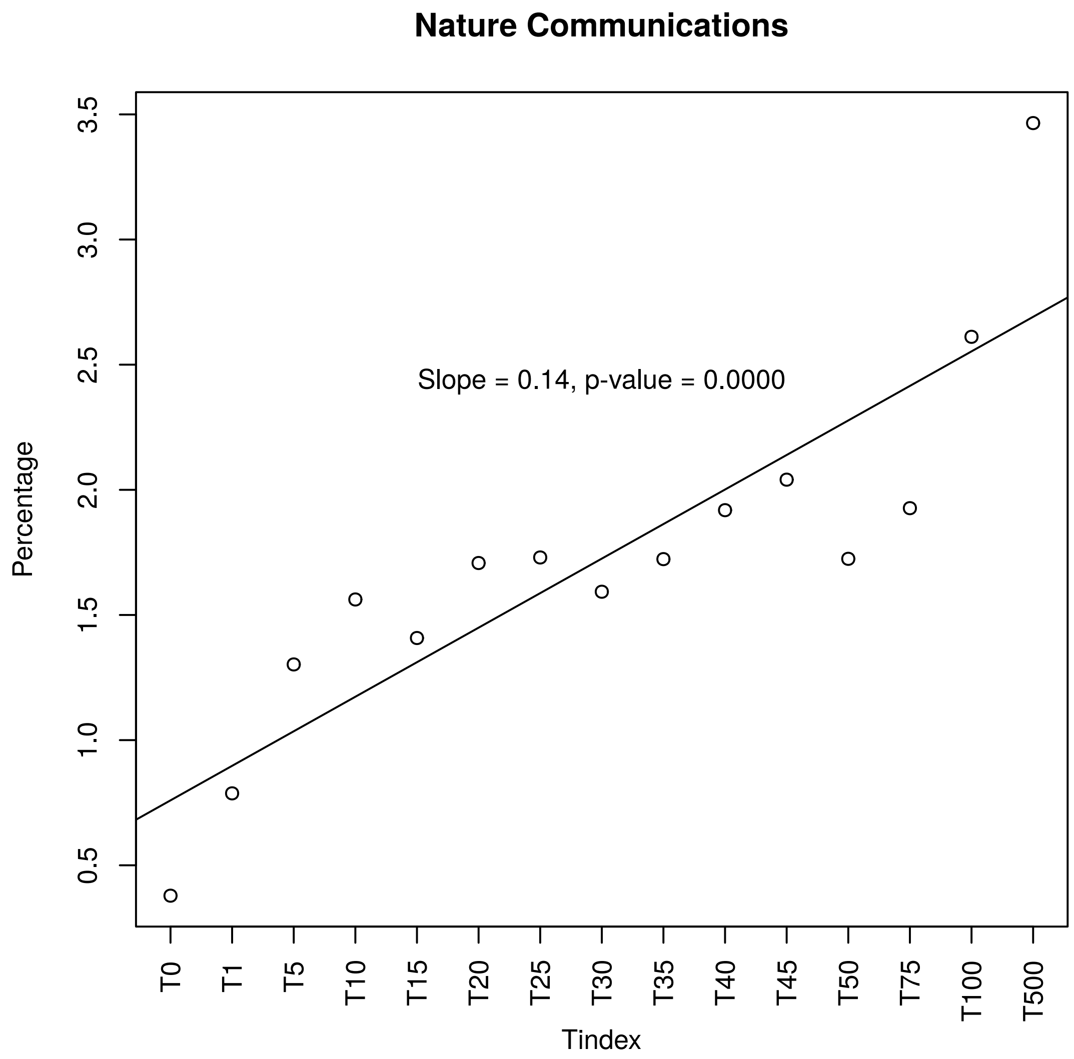

Supplementary Figure S3H

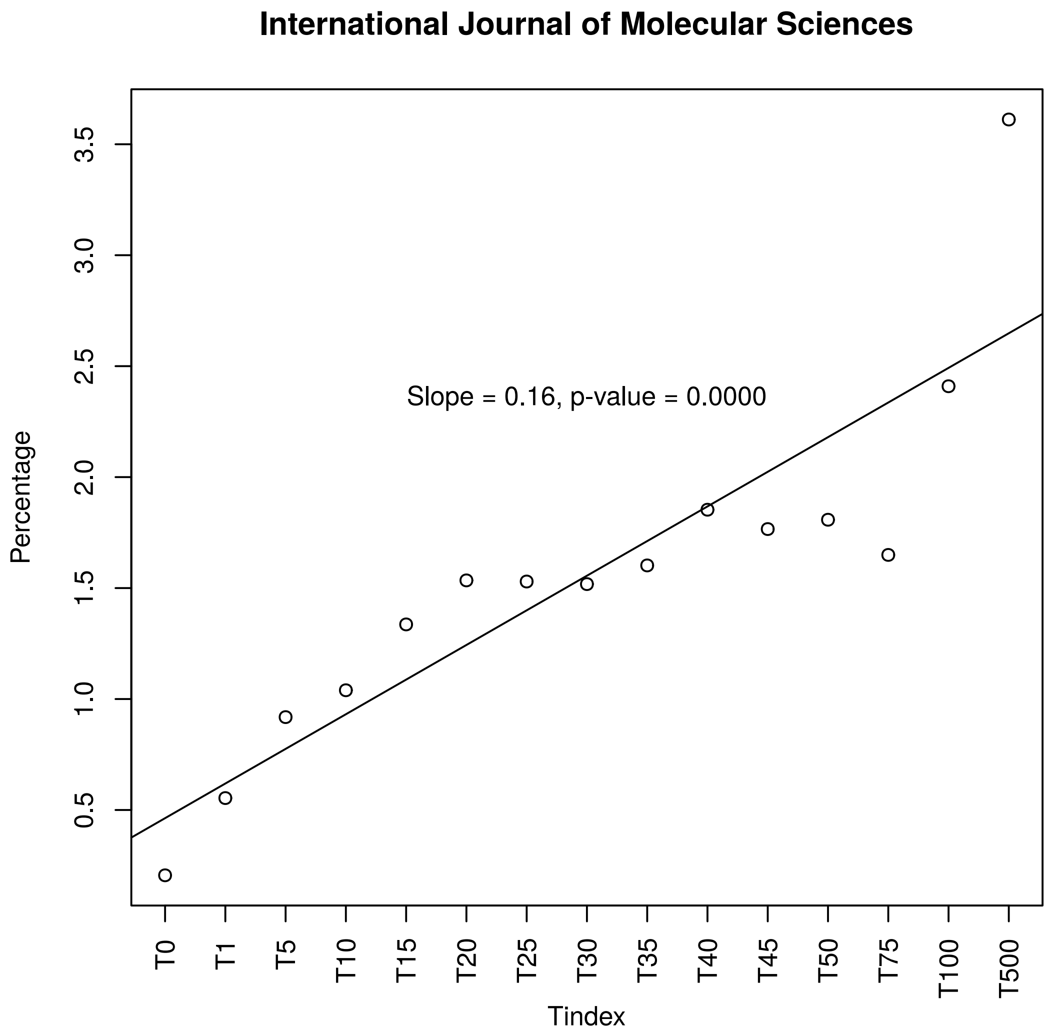

Supplement: Supplementary file 2 — Additional file 2. Figure S1. Explanation of variance of journals’ T-threshold data by principal coordinates. The graph illustrates how much of the data variance in the journals T-threshold vectors is explained by which principal coordinate. Figure S2. Loadings of principal coordinates PC1 and PC2. The loadings of principal coordinates PC1 and PC2 to the T-threshold coordinates (Tindex) are shown. Figure S3. Illustration of T-threshold vectors for selected journals together with indicative regression lines. The figures illustrate the T-threshold (Tindex) vectors for selected journals, the outliers in Figure 3 (four journals strong in T0, T1, … publications: “The Journal of Biological Chemistry”, “Molecular and Cellular Biology”, “The Journal of Cell Biology”, and four journals with overweight of T500, T100, T75, … publications: “PLOS One”, “Scientific Reports”, “Nature Communications” and “International Journal of Molecular Science”). Here, the y-axis value for a given T-threshold is the fraction of all T-threshold publications of the respective journal from the total pool of the same T-threshold publications in any journal (calculated in terms of FPEs). We also show a regression line as indicator of the trend along the T-threshold (Tindex) vectors together with the slope and the significance. Whereas the first four journals show a clear, significant decline towards T500, the four remaining ones exhibit a convincing rise towards higher T-thresholds. (A) The Journal of Biological Chemistry. (B) Molecular and Cellular Biology. (C) The Journal of Cell Biology. (D) Yeast. (E) PLOS One. (F) Scientific Reports. (G) Nature Communications. (H) International Journal of Molecular Science. [file 13062_2023_403_MOESM2_ESM.pdf]
